# Supplementary material for: The microbiome biomarkers of pregnant women’s vaginal area predict preterm prelabor rupture in Western China
Source: Front Cell Infect Microbiol. 2024 Oct 31;14:1471027. doi: 10.3389/fcimb.2024.1471027 (PMC11560878; doi:10.3389/fcimb.2024.1471027)
Supplement: Supplementary file 1 [file DataSheet1.zip › compare_1/Community/Pieplot/family/sample/C22.family.pieplot.pdf]

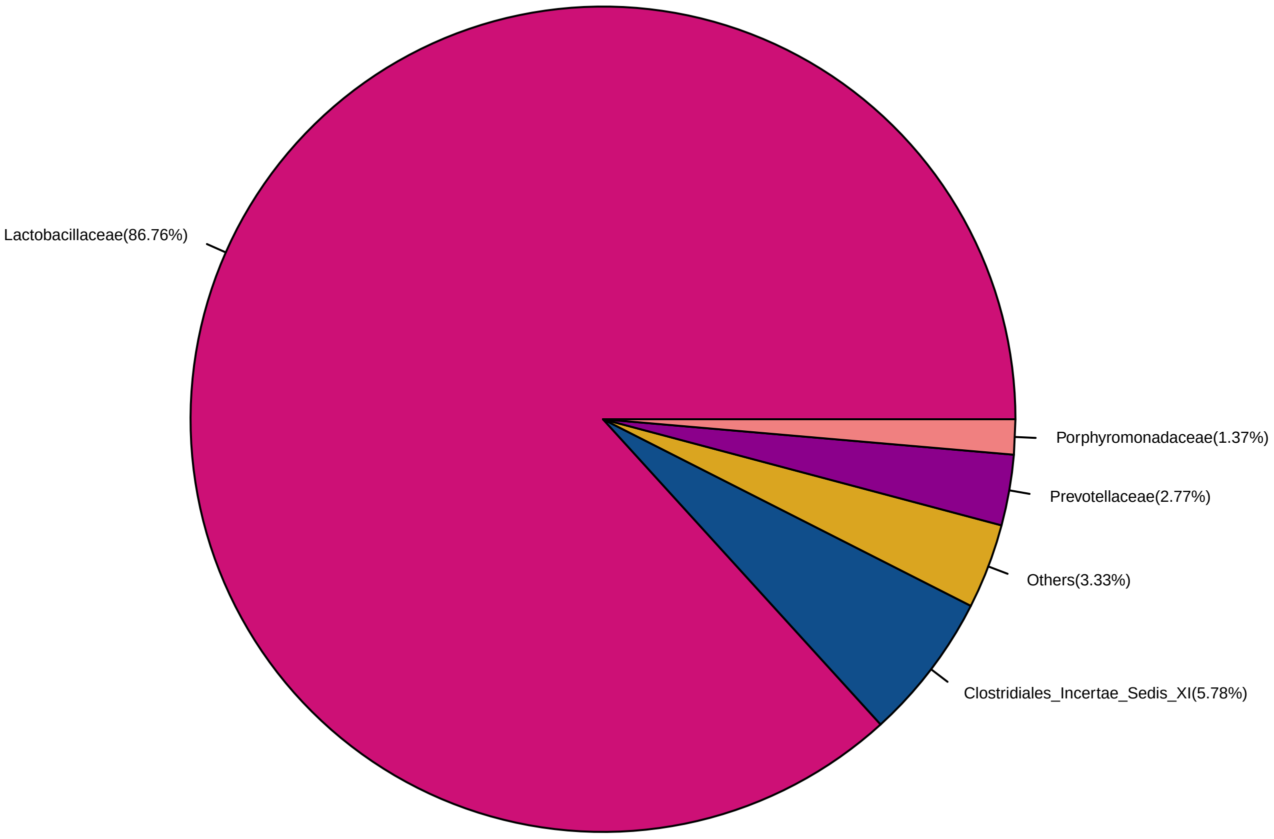
Lactobacillaceae(86.76%)
Porphyromonadaceae(1.37%)
Prevotellaceae(2.77%)
Others(3.33%)
Clostridiales_Incertae_Sedis_XI(5.78%)
